# A Localization Enhancement Method Based on Direct-Path Identification and Tracking for Future Networks

**DOI:** 10.3390/s25154538

**Published:** 2025-07-22

**Authors:** Yuhong Huang, Youping Zhao

**Affiliations:** School of Electronic and Information Engineering, Beijing Jiaotong University, Beijing 100044, China; yuhonghuang@bjtu.edu.cn

**Keywords:** direct path, localization, location tracking, non-line-of-sight (NLOS), range error, ultra-wideband (UWB)

## Abstract

Localization is one of the essential problems in the Internet of Things (IoT). Dynamic changes in the radio environment may lead to poor localization accuracy or discontinuous localization in non-line-of-sight (NLOS) scenarios. To address this problem, this paper proposes a localization enhancement method based on direct-path identification and tracking. More specifically, the proposed method significantly reduces the range error and localization error by quickly identifying the line-of-sight (LOS) to NLOS transition and effectively tracking the direct path. In a large testing hall, localization experiments based on the ultra-wideband (UWB) signal have been carried out. Experimental results show that the proposed method achieves a root mean square localization error of less than 0.3 m along the user equipment (UE) movement trajectory with serious NLOS propagation conditions. Compared with conventional methods, the proposed method significantly improves localization accuracy while ensuring continuous localization.

## 1. Introduction

High-precision localization for 6G is vital for a variety of new applications, such as industrial Internet of Things (IoT), health care, autonomous driving, digital twins, emergency response, and the tactile Internet [1,2,3]. The accuracy and reliability of localization techniques, particularly for indoor localization, have significantly improved, largely due to the increasing demand for location-based services in IoT applications [4].

In [5], Umer et al. reviewed several existing survey studies on localization, which compared different localization methods in terms of transmission bands, environment, and applications of localization. There are many indoor localization technologies based on radio signals, such as Bluetooth, Wi-Fi, and ultra-wideband (UWB). Based on different signal measurement technologies, these localization technologies can be divided into three main categories [6,7]: (i) power measurement-based methods, such as signal strength-based localization, (ii) angle measurement-based methods, such as angle of arrival (AOA) localization, and (iii) time measurement-based methods, such as time of arrival (TOA) and time difference of arrival (TDOA) localization. While localization methods of type (i) are cost-efficient, their localization accuracy is poor, particularly in non-line-of-sight (NLOS) scenarios. The reason is that the signal attenuation in these scenarios is only weakly correlated with propagation distance, leading to poor accuracy for range estimation. Methods of type (ii) can provide accurate estimation when the transmitter–receiver distance is small. However, even minor inaccuracies in angle calculation are translated into huge errors as the transmitter–receiver distance increases. Methods of type (iii) typically provide higher localization accuracy compared to methods of type (i) and (ii), but they generally rely on time synchronization and accurate direct-path identification. However, these methods are generally poor in localization accuracy or discontinuous localization in NLOS scenarios.

To address the above problem, some solutions have been proposed. For instance, machine learning (ML)-based methods for indoor localization have been developed, such as the feature-based deep long short-term memory network for UWB localization [8] and localization using convolutional neural networks (CNNs) [9]. In particular, a device-free activity detection and localization method based on CNN using channel state information measurement was proposed in [10] to improve the accuracy of WiFi-based localization. Recently, an interesting approach to UWB localization has been proposed by Gao et al. [11], who introduced a method based on CNN-SVM and a hybrid localization algorithm. Their method effectively addresses NLOS interference and enhances the accuracy of UWB localization. Recent advancements have also applied LSTM-based approaches to improve UWB positioning. Several representative studies demonstrate its effectiveness. For example, Gao et al. [12] proposed an LSTM-based regression model to predict UWB ranging errors by processing raw channel impulse response (CIR), achieving centimeter-level error prediction under NLOS conditions. However, practical environments pose challenges such as irregular signal observations, which affect prediction accuracy. In [13], the authors used an LSTM network to predict the positions of objects based on historical data from the UWB system and inertial navigation. Additionally, the integration of LSTM with other techniques has been explored. Yang et al. [14] combined CNN and LSTM to improve localization performance in NLOS scenarios, highlighting the importance of learning both spatial and temporal features. To mitigate NLOS effects, Kim et al. [15] combined LSTM with an extended Kalman filter (EKF), where LSTM was used to classify channel conditions and estimate ranging errors, and EKF was applied to correct those errors, enhancing positioning robustness. In [16], an LSTM neural network algorithm fused with the KF was presented to improve UWB positioning. Additionally, Wang et al. [17] demonstrated a cross-attention mechanism that fused received signal strength indication (RSSI) and distance signals to improve UWB localization in NLOS environments, incorporating LSTM for temporal feature extraction and position prediction. While ML-driven localization methods have shown improved accuracy to a certain extent in specific scenarios, it is time-consuming and labor-intensive to collect and label a massive amount of data. Fingerprint-based localization has attracted considerable attention due to the pervasive deployment of Wi-Fi infrastructures indoors. Zhu et al. [18] systematically reviewed an overview of the indoor localization technologies and systems based on wireless fingerprints presented in recent years, outlining their advantages, disadvantages, and underlying principles. Specifically, fingerprint-based localization determines user equipment (UE) location by building a fingerprint database to match location fingerprints [2]. However, this method requires labor-intensive database construction and maintenance processes, rendering it potentially impractical for dynamic or unexplored environments. To address performance degradation under environmental uncertainties, Zhang et al. proposed a variance-constrained local–global modeling method [19]. Notably, this method does not appear to have been evaluated in NLOS scenarios. A set of algorithms that estimate user positions using AoA values and the concept of the confidence region (CR) was presented in [4]. The confidence region defines the expected position uncertainty and helps to remove outlier measurements. Although these algorithms have been validated using a publicly available dataset, they should also be tested in real-world, larger experimental scenarios, since the AoA-based localization tends to produce large errors with increasing the transmitter-receiver distance. Schmidt et al. introduced a new representation of the channel impulse response (CIR) for single anchor localization systems, modeling the relationship between tag locations and the effective length of CIRs [20]. However, this method still lacks experimental validation to demonstrate improved localization performance. In [21], Wang et al. proposed a weighted constrained linear least squares algorithm based on path loss exponent estimation (PLE-WCLLS) for target localization in two-dimensional (2D) indoor wireless sensor networks under unknown transmission power conditions. However, in [21], the authors acknowledged that the proposed method may suffer from degraded performance due to the uneven anchor node distribution or severe multipath interference.

Despite progress in these methods, some existing studies lack measurement validation in the real world, especially in NLOS scenarios. More importantly, they do not fundamentally address the problem of direct-path delay estimation error in NLOS scenarios. In this paper, we propose a novel localization enhancement method based on direct-path identification and tracking. The main contributions of this paper are as follows:

- A novel localization enhancement method based on direct-path identification and tracking is proposed. The method can effectively track the direct path without requiring a large dataset, significantly reducing localization errors while ensuring continuous localization.
- A lightweight direct-path identification and tracking algorithm is developed. The line-of-sight (LOS) to NLOS transition can be identified in real time by monitoring abrupt changes in direct-path power (eliminating the need for training data or complex ML classifiers). The direct-path delay under NLOS conditions can be tracked using prior direct-path information, thereby reducing range error and further reducing localization error. The algorithm has a computational complexity of the order of O(n), enabling real-time execution even for resource-constrained IoT devices.
- Furthermore, in the proposed method, we further jointly reduce the localization errors by fusing the constrained estimated coordinates method, triangle centroid method, and localization base stations (BSs) preference.
- In a large testing hall, measurement experiments have been carried out to verify the effectiveness of the proposed scheme. A movable metal cabinet with the size of 1.3 m × 1 m × 2.5 m was used as a physical barrier to form about 23% NLOS propagation conditions. Experimental results show that the proposed method achieves a root mean square localization error of less than 0.25 m along the UE movement trajectory while ensuring continuous localization.

The remainder of this paper is organized as follows: Section 2 explains the proposed localization enhancement method and discusses some improvements to reduce range error and localization error; Section 3 presents a detailed analysis of the measurement experiments in a large testing hall; finally, Section 4 concludes the study.

## 2. Proposed Scheme

In a multi-base-station (BS) localization system, the distance between the BS (i.e., anchor node) and the UE (i.e., target node) is estimated based on propagation delay, which is then used to solve the localization equation to obtain the UE location. Based on the general localization procedure, the proposed scheme consists of three steps in this paper, as follows. The proposed scheme can be applied to both the single-input-single-output (SISO) system and the multiple-input-multiple-output (MIMO) system. Without loss of generality, we consider a SISO uplink where both the BS and UE are equipped with an omnidirectional antenna.

### 2.1. Step 1: Identification of Direct Path

First, the direct path needs to be identified. In general, under the line-of-sight (LOS) scenario, the strongest path between the transmitter and the receiver can be identified as the direct path. However, direct-path delay estimation errors become significant when identifying the strongest path as the direct path under the NLOS scenario, where the LOS path between the UE and the BS is obstructed by physical barriers. To mitigate this issue, the earliest arrival path can be tentatively identified as the direct path within a wider frequency band by changing the channels. The reason is that multipath signals operating at different frequencies suffer varying levels of propagation attenuation. The formula can be written as

(1) $$t_{DP\_nlos}=\underset{t_{m,i}}{\arg\min}\left\{t_{m,i}|P_{m,i}>P_{th},m=1,2,...,M\right\},$$

where $t_{DP\_nlos}$ is the direct-path time of arrival for the NLOS scenario, *M* is the number of channels, $t_{m,i}$ is the time of arrival of the *i*-th path in channel *m*, $P_{m,i}$ is the power of the *i*-th path in channel *m*, and $P_{th}$ is the predefined threshold. The predefined threshold is determined as follows. Firstly, the CIR data can be obtained after the baseband data undergoes filtering and correlation processing. Then, the power of the strongest path in the CIR data is calculated in dB. We set $P_{th}$ to be 15 dB below the power of the strongest path. The first peak whose power exceeds the threshold is identified as the direct path at that moment. If no peak is detected, the threshold is adjusted accordingly.

**Figure 1:**
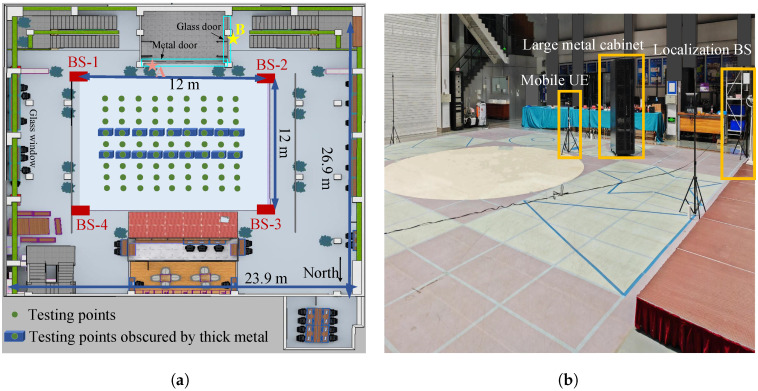
The testing hall scenario: (**a**) Floor plan of the testing hall. (**b**) Three-dimensional view of the testing hall.

**Figure 2:**
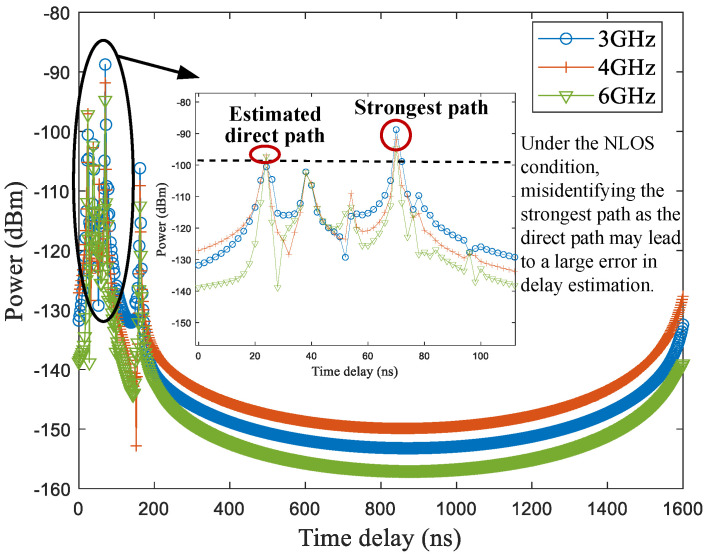
Feasibility analysis: the direct path can be identified within a wider frequency band by changing the channel.

A testing hall scenario is shown in Figure 1a, which will be introduced in detail in Section 3. The feasibility analysis of identifying the direct path within a wider frequency band by changing the channel is illustrated in Figure 2. The transmitted signal is a wideband signal operating at different center frequencies, including 3 GHz, 4 GHz, and 6 GHz. The delay resolution is 2 ns, and the transmit power is 0 dBm. The transmitter and the receiver are located at points A and B, respectively, as marked in Figure 1a. The distance between the transmitter antenna and the receiver antenna is 5.22 m, corresponding to a true direct-path propagation delay of 17.40 ns. Due to the obstruction by a metal door and a glass door between the transmitter and the receiver, there exists an NLOS propagation condition. As shown in Figure 2, multipath attenuation varies across different channels. Under the NLOS propagation condition, the strongest path is not the direct path. In this case, it may cause a large delay estimation error if the strongest path is incorrectly identified as the direct path. Under the three tested conditions with different center frequencies, the strongest path appears at around 70 ns, resulting in an estimated delay error of approximately 52.6 ns. Fortunately, when changing the channel, the delay corresponding to the earliest arrival path is 24 ns, leading to a smaller direct-path delay estimation error of 6.6 ns. This delay error may be further reduced by selecting more suitable channels, thereby decreasing the range error. For example, the IEEE 802.15.4/4z standards define the 16 channels for UWB signal, where each channel is a combination of a center frequency and a maximum bandwidth [22,23].

### 2.2. Step 2: Tracking of Direct Path

Since the estimated direct-path delay error under the NLOS scenario is notably larger than that under the LOS scenario, it is particularly important to focus on accurate direct-path tracking under the NLOS scenario. Therefore, it is necessary to effectively identify the transition from LOS to NLOS. In this paper, such a transition can be identified once the direct path changes abruptly. Specifically, if the change of the direct-path power at the current moment compared to that at the previous moment is greater than a set threshold, the direct path is considered to have changed abruptly. This set threshold can be determined through statistical analysis of measurement data collected in an environment. Specifically, we can collect CIR data under the LOS scenario, and then compute the statistical confidence interval (e.g., 90%) of the direct-path power variation between consecutive time steps. Then, the threshold can be determined by applying a safety margin above this interval. Unlike most existing ML-based methods that rely on extensive training data for LOS/NLOS classification [24,25,26], our approach eliminates the need for offline model training.

**Figure 3:**
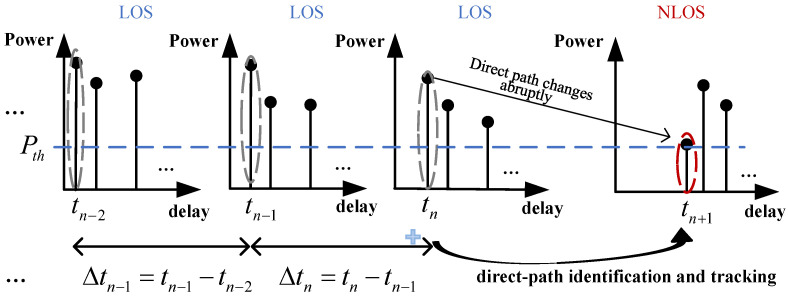
Direct-path identification and tracking.

As shown in Figure 3, direct-path tracking is described as the direct path under the identified NLOS propagation condition at the present localization moment can be predicted based on the LOS propagation conditions at its previous *n* localization moments. The value of *n* is typically kept small (usually fewer than 10 localization moments). This is primarily due to two critical considerations: first, to avoid accumulating errors during the direct-path tracking process, and second, to reduce system complexity. Considering the worst case where few LOS conditions are available in the scenario, the tracked direct path at this localization moment in the NLOS can serve as prior information for tracking the direct path under the NLOS scenario later.

The direct-path tracking is expressed as

(2) $$t_{n+1}=t_{n}+\sum_{i=1}^{n}\omega_{i}\left(\frac{\Delta t_{i}\cdot c\cdot \cos\left(\arcsin\left(\Delta h/\left(\Delta t_{i}\cdot c\right)\right)\right)}{v_{i}}\right),$$

(3) $$\omega_{i}=\frac{e^{-\lambda (n-i)}}{\sum_{i=1}^{n}e^{-\lambda (n-i)}},$$

where $t_{n+1}$ is the direct-path delay at the (n+1)-th localization (i.e., the current localization moment), $t_{n}$ is the direct-path delay at the *n*-th localization, $\Delta t_{i}$ ($\Delta t_{i}=t_{i}-t_{i-1},i=1,...,n$) is the difference between direct-path delay at the *i*-th localization and (i−1)-th localization; *c* is the speed of light in free space, $v_{i}$ is the UE’s moving speed at the *i*-th localization, which can be conveniently captured via the built-in sensors of IoT devices; and Δh is the height difference between the transmitter and the receiver. In practical deployment scenarios, the location of the localization BS (i.e., the transmitter) remains constant, and the height variation of the UE is relatively small. For example, wearable or vehicle-mounted UEs generally maintain an essentially fixed elevation. If needed, built-in inertial sensors (e.g., an inertial measurement unit (IMU)) could be used to measure this parameter, although they are not required in our current implementation. $\omega_{i}$ is the weight at the *i*-th localization, which indicates the influence of the previous direct-path delays on the tracking of $t_{n+1}$, and λ is a parameter governing the exponential decay rate. We compute the partial derivative $\frac{\partial \omega_{i}}{\partial \lambda }=\omega_{i}\left(\sum_{k=1}^{n}\left(n-k\right)\omega_{k}-\left(n-i\right)\right)$. Analysis reveals that:, as λ→0, the weights $\omega_{i}$ follow a uniform distribution, indicating strong predictability of the current direct-path delay from historical data. This regime suits stable linear trajectories. When λ→1, recent direct-path delays dominate the weighting, enabling rapid adaptation to nonlinear or dynamic movements. Based on experimental validation, we empirically set λ∈[0,0.5] when the UE’s movement trajectory is linear, and λ=1 otherwise.

The meaning of Equation (2) is explained in detail below. $\Delta t_{i}$ represents the difference between direct-path delay at the *i*-th localization and (i−1)-th localization, so $\Delta t_{i}\cdot c$ is the change in direct-path propagation distance. The elevation angle of the direct path relative to the horizontal plane can be calculated by the term $\arcsin(\Delta h/\left(\Delta t_{i}\cdot c\right))$. The term $\cos(\arcsin\left(\Delta h/\left(\Delta t_{i}\cdot c\right)\right))$ computes the ratio of the horizontal distance change component to the total slant distance change ($\Delta t_{i}\cdot c$). This calculation helps mitigate the impact of height differences on the delay. Hence, (Δti·c·cos(arcsin(Δh/(Δti·c))))(Δti·c·cos(arcsin(Δh/(Δti·c))))vivi represents the time increment when the user equipment (UE) is moving at speed $v_{i}$, directly impacting $\omega_{i}$. By accounting for both Δh and $v_{i}$, the prediction of $t_{n+1}$ can be better aligned with real-world scenarios, resulting in a more accurate estimation.

The proposed direct-path identification and tracking is presented in Algorithm 1.

Equations (2) and (3) involve only *n* exponential operations and summations, resulting in a computational complexity of O(n). This efficiency of the tracking enables real-time execution even for resource-constrained IoT devices.
**Algorithm** **1**: Proposed direct-path identification and tracking1:**for** each localization moment **do**2:    identify the transition from LOS to NLOS3:    **if** the direct path does not change abruptly **then**    ▹Under the LOS scenario4:        identify the earliest arrival path as the direct path5:    **else**                      ▹Under the NLOS scenario6:        **if** there are fewer than three consecutive moments under LOS **then**7:           calculate the direct-path delay using Equation (1)8:        **else**9:           **for** i=1,...,n **do**10:               obtain Δh and $v_{i}$11:               calculate $\Delta t_{i}=t_{i}-t_{i-1}$12:               calculate $\omega_{i}=\frac{e^{-\lambda (n-i)}}{\sum_{i=1}^{n}e^{-\lambda (n-i)}}$13:               calculate the direct-path delay using Equation (2) ▹ Track the direct path14:           **end for**15:        **end if**16:    **end if**17:**end for**

### 2.3. Step 3: Obtain Localization Coordinates

In Step 2, the estimated direct-path delay between the base station and the UE can be obtained. Then, the estimated range can be calculated using the estimated direct-path delay, i.e., the estimated range is the product of the delay and the radio propagation velocity (i.e., the speed of light in free space). Finally, the UE coordinates can be obtained by solving localization equation sets based on the estimated range.

In this subsection, several different geometrical situations corresponding to localization errors caused by various reasons such as NLOS are visually presented. Note that the localization errors caused by range errors can be mitigated based on Step 2.

**Figure 4:**
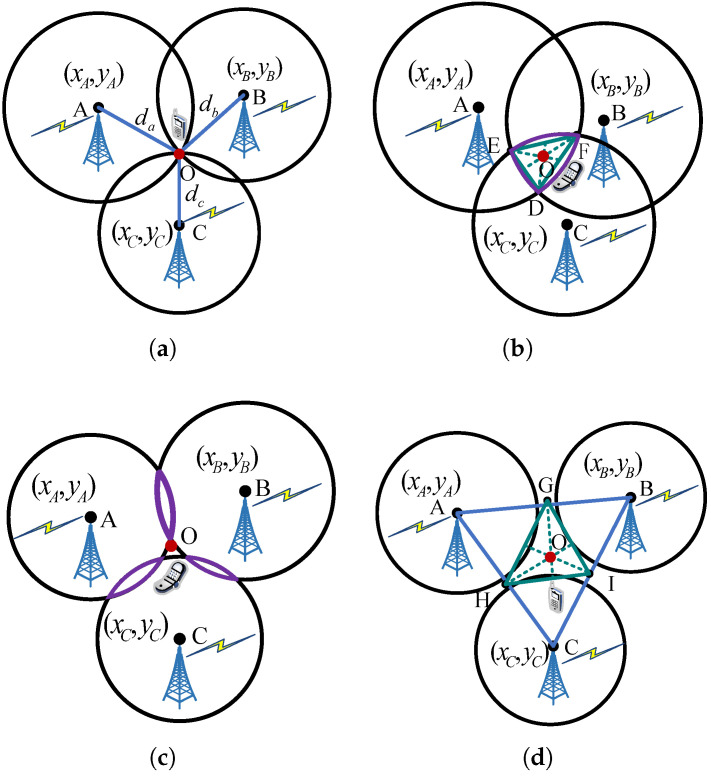
Geometrical situations that may have an impact on the localization error: (**a**) Case 1: normal. The three circles intersect at one point when three BSs have no range error. (**b**) Case 2: the three circles intersect a common area when one of the BSs has a range error. (**c**) Case 3: the three circles intersect an area two by two when more than one of the BSs has a range error. (**d**) Case 4: the three circles do not intersect with each other when more than one of the BSs has a range error.

As shown in Figure 4, points A, B, and C are the centers of the circles, representing the localization BSs involved in the solution equation. The coordinates of these BSs are denoted as $\left(x_{j},y_{j}\right)$, where j∈{A,B,C}. The radii $d_{a}$, $d_{b}$, and $d_{c}$ are the estimated ranges between the BSs and the UE based on their respective direct-path delays. Point O denotes the estimated UE location, which can be represented by $\left(\hat{x},\hat{y}\right)$.

Ideally, when the estimated ranges of the three BSs are error-free, the three circles intersect at a single point, as illustrated in Figure 4a. In this case, the estimated UE coordinates $\left(\hat{x},\hat{y}\right)$ can be determined based on trilateration [7], which can be formulated as

(4) $$\left\{\begin{array}{c} \sqrt{{\left(\hat{x}-x_{A}\right)}^{2}+{\left(\hat{y}-y_{A}\right)}^{2}}=d_{a},\\ \sqrt{{\left(\hat{x}-x_{B}\right)}^{2}+{\left(\hat{y}-y_{B}\right)}^{2}}=d_{b},\\ \sqrt{{\left(\hat{x}-x_{C}\right)}^{2}+{\left(\hat{y}-y_{C}\right)}^{2}}=d_{c}. \end{array}\right.$$

However, several factors may introduce localization errors, causing the circles to no longer intersect at a single point. Therefore, it is necessary to improve the localization algorithm to mitigate the localization error. In the proposed scheme, we further jointly reduce the localization errors by fusing the constrained estimated coordinates method, triangle centroid method, and localization BSs preference. The following situations may cause localization errors.

(1) Estimated coordinates are anomalous.

The estimated UE movement trajectory occasionally has anomalous results because of localization discontinuity, such as the cases of burr and through the wall. The estimated localization coordinates deviate from the solution area of the localization BSs under the cases.

To address this, we impose a geometric constraint on the solution space. The localization solution area of the serving BSs can be used as a constraint condition, which can be represented as

(5) $$\left\{\begin{array}{c} \min\left(x_{A},x_{B},x_{C}\right)<\hat{x}<\max\left(x_{A},x_{B},x_{C}\right),\\ \min\left(y_{A},y_{B},y_{C}\right)<\hat{y}<\max\left(y_{A},y_{B},y_{C}\right), \end{array}\right.$$

where $\left(\hat{x},\hat{y}\right)$ denotes the estimated UE coordinates. With the aid of this method, a certain degree of correction can be applied to the horizontal or vertical coordinates of the anomalous estimated coordinates.

(2) The localization equation cannot be solved to obtain the estimated coordinates.

When the BSs have large range errors caused by the very imprecise estimated delay of the direct path, the localization equation cannot be solved to obtain the UE coordinates. As shown in Figure 4b–d, three circles intersect in an area or completely do not intersect with each other, making it difficult to determine the localization coordinates.

For the case of Figure 4b, the triangle centroid method [27] can be used to reduce the localization error. The coordinates of intersection points D, E, and F can be obtained on the basis of Equation (4), represented as $\left(x_{D},y_{D}\right)$, $\left(x_{E},y_{E}\right)$, and $\left(x_{F},y_{F}\right)$, respectively. Then, the centroid of ΔDEF can be obtained. For example, the coordinates of the intersection point D can be obtained by the following equation

(6) $$\left\{\begin{array}{c} \sqrt{{\left(x_{D}-x_{A}\right)}^{2}+{\left(y_{D}-y_{A}\right)}^{2}}=d_{a},\\ \sqrt{{\left(x_{D}-x_{B}\right)}^{2}+{\left(y_{D}-y_{B}\right)}^{2}}=d_{b},\\ \sqrt{{\left(x_{D}-x_{C}\right)}^{2}+{\left(y_{D}-y_{C}\right)}^{2}}\leq d_{c}. \end{array}\right.$$

Similarly, the coordinates of the intersection points E and F can be obtained using Equation (6). Therefore, the UE coordinates $\left(\hat{x},\hat{y}\right)$ can be represented by

(7) $$\left(\hat{x},\hat{y}\right)=\left(\frac{x_{D}+x_{E}+x_{F}}{3},\frac{y_{D}+y_{E}+y_{F}}{3}\right).$$

For the cases of Figure 4c,d, the points G, H, and I could be selected according to the calculation of length ratio to obtain their coordinates. The coordinates of points G, H, and I are expressed as $\left(x_{G},y_{G}\right)$, $\left(x_{H},y_{H}\right)$, and $\left(x_{I},y_{I}\right)$, respectively. Then, the triangle centroid method [27] can be used to obtain the UE coordinates based on Equation (7). For example, the coordinates of point G can be represented by

(8) $$\left\{\begin{array}{c} x_{G}=x_{A}+\frac{d_{a}\left(x_{B}-x_{A}\right)}{d_{a}+d_{b}},\\ y_{G}=y_{A}+\frac{d_{a}\left(y_{B}-y_{A}\right)}{d_{a}+d_{b}}. \end{array}\right.$$

Similarly, the coordinates of points H and I can be obtained using Equation (8). Then, the UE coordinates $\left(\hat{x},\hat{y}\right)$ can be expressed as

(9) $$\left(\hat{x},\hat{y}\right)=\left(\frac{x_{G}+x_{H}+x_{I}}{3},\frac{y_{G}+y_{H}+y_{I}}{3}\right).$$

A common goal across these cases of Figure 4b–d is to determine the intersection points of the intermediate triangular regions covered by the localization BSs. Therefore, if the coordinates of these intersection points can be calculated using Equation (6), then the UE coordinates can be obtained using Equation (7). Otherwise, the intersection coordinates of points can be calculated using Equation (8), and then, the UE coordinates can be estimated using Equation (9). These processes are fully automated to ensure algorithmic efficiency during the localization process.

Since the proposed method relies on reducing range errors by direct-path tracking to enhance localization, the UE coordinates are determined by solving localization equations based on trilateration using the estimated range. As a result, our method generally requires at least three BSs to ensure accurate localization.

If there are more than three localization BSs in the localization system, it is necessary to optimally select the serving BSs for localization to enhance localization performance. A smaller geometric dilution of precision (GDOP) indicates a better geometric configuration and generally results in a smaller localization error. Therefore, the localization BS with a smaller GDOP can be selected to participate in the localization solution. The GDOP can be calculated by

(10) $$V_{\mathrm{GDOP}}=\sqrt{tr(G^{T}G^{-1})},$$

where *G* denotes the coefficient matrix of the localization system of linear equations built from the estimated ranges between the BSs and the UE, and tr() is the trace of the matrix [28,29].

### 2.4. Comparative Analysis

To ensure that the proposed method aligns with the latest developments, we reviewed recent specifications and standards related to UWB localization, including documents and recent updates from the IEEE Standards Association and the FiRa Consortium.

The most up-to-date specifications from the FiRa Consortium are the FiRa Core 3.0 Specifications, while the relevant IEEE standards include IEEE 802.15.4-2020 and the draft amendment under development by IEEE Task Group 4ab (TG4ab). A brief comparison of representative items between the proposed method and these standards is presented in Table 1.

## 3. Experimental Results

In this section, we describe the measurement experiments that have been carried out to verify the effectiveness of the proposed scheme. Baseline method-1 is trilateration based on two-way ranging [7], a widely adopted approach in engineering applications, which estimates UE locations by solving localization equations using estimated ranges. Baseline method-2 is a commonly used localization method based on a long short-term memory (LSTM) network [35], trained on existing datasets (e.g., CIR data containing rich dynamic channel features) to directly estimate UE coordinates.

### 3.1. Testing Scenario

The measurement scenario is a large testing hall with dimensions 23.9 m × 26.9 m × 8 m, as shown in Figure 1. The testing hall contains concrete walls, glass doors and windows, wooden furniture, and large metal equipment, among other materials. The selected testing area is a square field with dimensions 12 m × 12 m. Four localization BSs with antenna heights of 1.66 m are placed at the four corners of the testing area. The height of the UE antenna is 1.24 m. The spacing of the 81 testing locations along the UE movement trajectory is 1 m. In order to create both LOS and NLOS propagation conditions for the experiment, a movable metal cabinet with dimensions 1.3 m × 1 m × 2.5 m is used as a physical barrier to form about 23% NLOS propagation conditions. Specifically, the number of locations occluded by the metal cabinet is 18, and the metal cabinet is moved to block the LOS path between the UE and the localization BS when the UE is moving. In the measurement experiments, the moving speed of the UE is 1 m/s, and the positioning frequency of the UE is set to 1 Hz. These values were chosen specifically for automated testing.

The experimental localization BSs and the UE are both DW1000 chip-based devices [36]. The transmitted signal is an UWB signal complying with the IEEE 802.15.4 standard [22], operating at a center frequency of 3993.6 MHz and a bandwidth of 499.2 MHz. The transmit power of the UWB signal is −41.3 dBm/MHz. The resolution of CIR data is about 1 ns.

**Figure 5:**
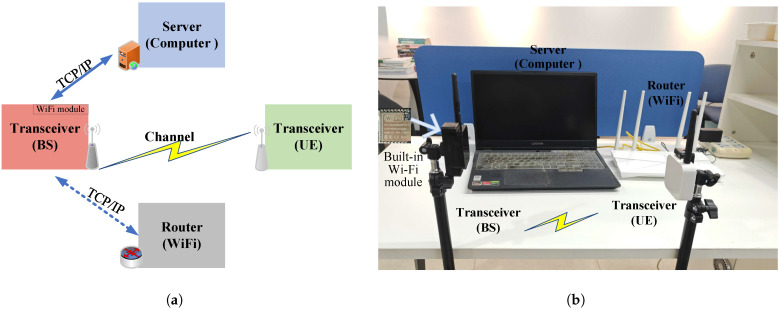
Architecture of the real-time measurement and localization system: (**a**) Block diagram of the system. (**b**) Photograph of the hardware platform.

The architecture of the real-time measurement and localization system is shown in Figure 5. The Transmission Control Protocol/Internet Protocol (TCP/IP) transport protocol is used to collect measurement data. Table 2 lists the major experimental parameters in the measurement experiments.

### 3.2. Actual Experimental Results

**Figure 6:**
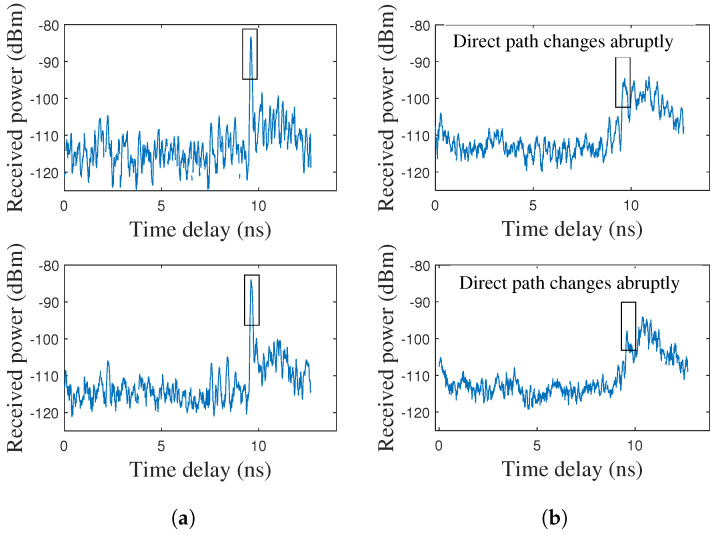
Measured CIR under LOS and NLOS scenarios: (**a**) Examples of LOS CIR. (**b**) Examples of NLOS CIR.

In the experiments, the representative examples of the measured CIR under the LOS and NLOS scenarios between the localization BS and the UE are shown in Figure 6. It can be seen that under the LOS scenario, there is a prominent peak along the rising edge at the direct path, and the peak is the strongest path. Under the NLOS scenario, the peak along the rising edge is weaker, and the direct path is generally not the strongest path. Therefore, it may cause large errors in the direct-path delay estimation if the strongest path is always identified as the direct path under the NLOS scenario. Fortunately, the proposed method can avoid this situation by effectively identifying and tracking the direct path. The transition from LOS to NLOS can be effectively identified once the direct path changes abruptly.

**Figure 7:**
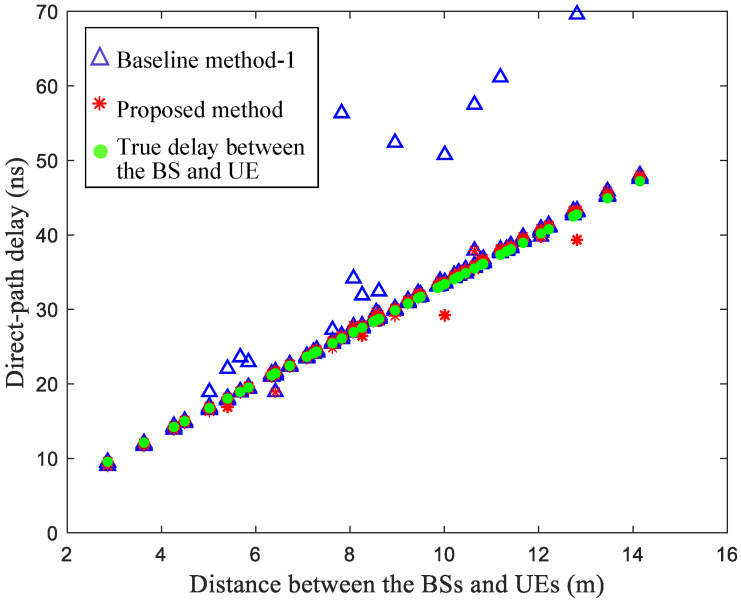
Comparison of the estimated direct-path delay when using the baseline method-1 and the proposed method under 23% NLOS propagation conditions along the UE trajectory.

Figure 7 compares the results of the estimated direct-path delays between the localization BSs and the UEs when using the baseline method-1 and the proposed method. The direct-path delay under the identified NLOS scenario would be effectively tracked using the proposed method, where the estimated direct-path delay is closer to the true delay along the UE movement trajectory. When using the proposed method, the mean absolute error of the estimated direct-path delay along the UE movement trajectory is about 1.17 ns. However, the baseline method-1 does not track the direct path under the NLOS scenario. When using the baseline method-1, the mean absolute error of the estimated direct-path delay along the UE movement trajectory is about 3.16 ns. A larger delay error generally leads to a larger range error and localization error.

**Figure 8:**
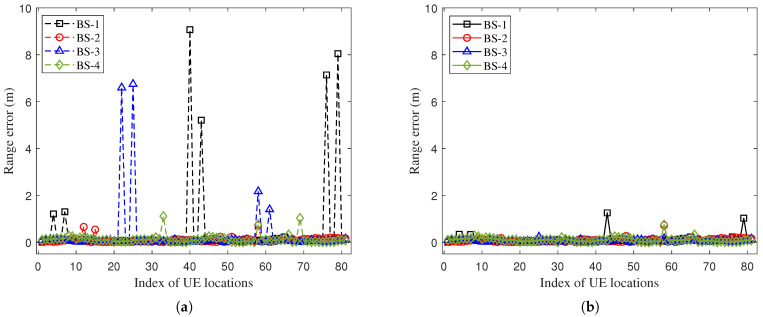
Comparison of range errors at different testing locations under 23% NLOS propagation conditions along the UE trajectory: (**a**) Range error when using the baseline method-1. (**b**) Range error when using the proposed method.

The range-error results of the proposed method and the baseline method-1 are shown in Figure 8. Due to the fact that some testing points are completely blocked by a large metal cabinet, the transmitted signal might be reflected multiple times by other objects and arrive at the UE through NLOS paths. The maximum range error of baseline method-1 is about 9 m, while the maximum range error of the proposed method is reduced to 1 m. To evaluate the range accuracy, we calculate the mean absolute range error. The mean absolute range error of the baseline method-1 is 0.95 m, while that of the proposed method is 0.35 m. The proposed method significantly improves the range accuracy due to its effective tracking of the direct-path delay under the NLOS scenario, and the range accuracy is improved by 63.0%.

**Figure 9:**
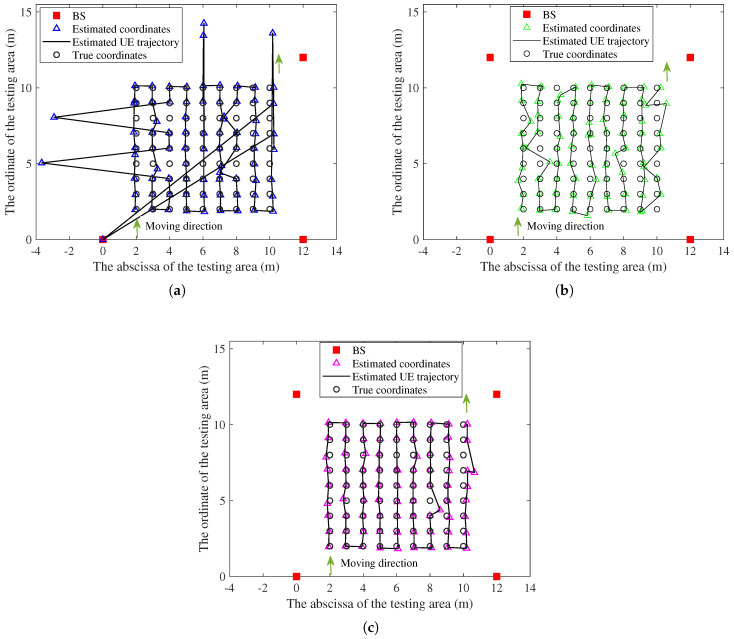
Comparison of localization trajectories in the testing hall under 23% NLOS propagation conditions along the UE trajectory: (**a**) Localization results of the baseline method-1. (**b**) Localization results of the baseline method-2. (**c**) Localization results of the proposed method.

Figure 9 presents the localization trajectories of the proposed method and the baseline method-1. When using the baseline method-1, there are some anomalies along the estimated trajectory of the UE, with these estimated anomaly coordinates significantly deviating from the true coordinates, causing discontinuous localization multiple times. The estimated locations of the proposed method generally maintain continuous alignment with the movement trajectory of UE.

**Figure 10:**
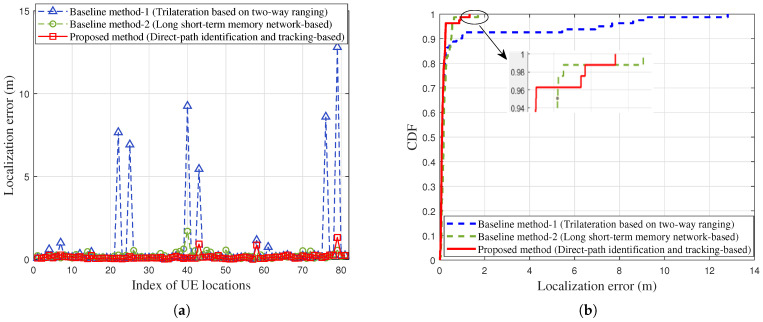
Comparison of localization error when using the baseline methods and the proposed method under 23% NLOS propagation conditions along the UE trajectory: (**a**) Localization errors at different testing locations. (**b**) The CDF of localization errors.

The localization errors of the proposed method and baseline methods are shown in Figure 10. We also compare the proposed method with a commonly used localization method based on a long short-term memory (LSTM) network, which is labeled baseline method-2. Table 3 lists the major parameter configurations for the used LSTM model (baseline method-2). As can be seen from Figure 10a, due to the 23% NLOS propagation conditions caused by thick metal obstructions, there are significant localization errors at certain testing locations for both baseline methods, especially for the baseline method-1. For baseline method-2, since it relies on a model trained using existing datasets to estimate UE locations, its localization results lack interpretability. Figure 10b shows the cumulative distribution function (CDF) curve of localization error under the three methods. It can be seen that the proposed method achieves better localization performance. To evaluate the localization accuracy of these methods, we calculate the root mean square error (RMSE) of localization error along the UE movement trajectory. The RMSE localization errors for the two baseline methods are approximately 2.39 m and 0.32 m, respectively. For the proposed method, the RMSE localization error is about 0.24 m, representing reductions of 89.9% and 25% compared with the two baseline methods, respectively. However, in multiple experiments, we found that the localization performance of baseline method-2 may be worse when the training dataset is changed (e.g., when the order of the training dataset is disrupted). In addition, we also compare the mean error, maximum error, and 90% error of localization performance between the three methods, as shown in Table 4. The results show that the proposed method demonstrates lower localization errors across the four error metrics than the two conventional methods. In summary, the proposed method shows better localization performance and also offers certain interpretability, since it can identify and track the direct path effectively.

**Figure 11:**
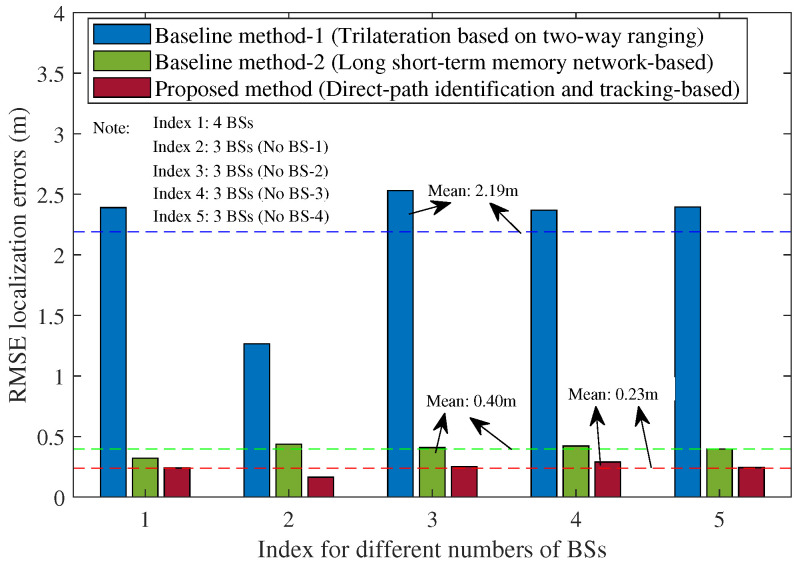
Comparison of the RMSE localization error when using the baseline methods and the proposed method under different numbers of BSs, with 23% NLOS propagation conditions along the UE trajectory (Note: The dotted lines represent the average RMSE localization errors of these methods).

**Figure 12:**
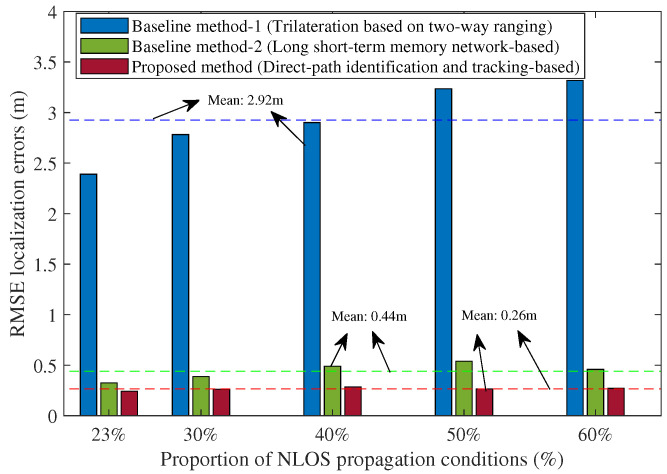
Comparison of the RMSE localization error when using the baseline methods and the proposed method under different proportions of NLOS propagation conditions along the UE trajectory (Note: The dotted lines represent the average RMSE localization errors of these methods).

In addition, to further demonstrate the robustness of the proposed method under different testing conditions, we also compared the results of different numbers of BSs and different proportions of NLOS propagation conditions, as shown in Figure 11 and Figure 12. In Figure 11, each index corresponds to a different number of BSs in various cases. For example, Index 1 (3 BSs, No BS-1) means that there are 3 BSs with BS-1 removed in the testing hall scenario of Figure 1. The results in Figure 11 indicate that the proposed method consistently outperforms the baseline methods in all cases even with fewer BSs. The averages of the RMSE localization errors for the two baseline methods are approximately 2.19 m and 0.4 m, respectively. For the proposed method, the average RMSE localization error is about 0.23 m. Notably, in the case with three BSs (with BS-1 removed), the average RMSE localization error is slightly lower than the case with four BSs, potentially due to the fact that BS-1 was located near glass window walls on the east and south sides. In the scenario shown in Figure 12, the averages of the RMSE localization errors for the two baseline methods are approximately 2.92 m and 0.44 m, respectively. For the proposed method, the average RMSE localization error is about 0.26 m. It can be seen that our method consistently and significantly outperformed both comparison methods across all the cases of different proportions of NLOS propagation conditions, even under 60% NLOS propagation conditions along the UE trajectory.

## 4. Conclusions

In this paper, a localization enhancement method based on direct-path identification and tracking is proposed. The localization performance can be effectively improved by using the proposed method, especially in the NLOS scenarios. Experimental results show that the proposed method can achieve the root mean square localization error of less than 0.3 m in a testing hall scenario even with serious NLOS. In future work, we will continue to validate the effectiveness of the proposed method in complex industrial scenarios with various types of interference, and also continue to optimize the complexity of the proposed method.

## Figures and Tables

**Table 1:** Comparison of different standards.

Items	IEEE Standards Association [22,23,30,31]	FiRa Consortium [32,33,34]	Proposed Method	Consistent with IEEE/FiRa?
Localization domain	Primarily in time domain	Primarily in time domain	Time domain	Yes
Direct-path identification	Primarily use the strongest path	Recommend the earliest path	Select the earliest arrival path; track direct path under NLOS	Yes
Preference for algorithmic overhead	Low power; low complexity	Low power; lightweight computing	Low complexity; lightweight computing	Yes
Adaptability/scalability	Open protocol	Specifications & certification based on IEEE	IEEE-based; FiRa-compatible	Yes
Purpose/vision of latest standard	TG4ab: enhancing UWB PHY/MAC, including ranging techniques	FiRa Core 3.0: enable precise location awareness	Reduce the ranging & localization errors, especially under NLOS	Yes

**Table 2:** Experimental parameters.

Parameter	Value/Explanation
Center Frequency	3993.6 MHz
Bandwidth	499.2 MHz
Transmit Power of BS	−41.3 dBm/MHz
Resolution of CIR data	1 ns
Antenna Configurations of BS	An omnidirectional antenna
Antenna Configurations of UE	An omnidirectional antenna
Height of the BS Antenna	1.66 m
Height of the UE Antenna	1.24 m
Speed of Light in Free Space	$3\times 10^{8}$ m/s
Measurement Scenario	A large testing hall, dimensions 23.9 m × 26.9 m × 8 m
Testing Area	A square field, 12 m × 12 m
A Movable Metal Cabinet	1.3 m × 1 m × 2.5 m

**Table 3:** Major parameter configurations for the used LSTM model (baseline method-2).

Parameter	Value
LSTM Layer	5
Max Epochs	1200
Mini-Batch Size	30
Initial Learn Rate	0.001
Learn-Rate Drop Period	800
Learn Rate Drop Factor	0.5

**Table 4:** Comparison and analysis of localization performance when using different methods under 23% NLOS propagation conditions along the UE trajectory.

Methods	Root Mean Square Error (m)	Mean Error (m)	Maximum Error (m)	90% Error (m)
Baseline Method-1	2.39	0.78	12.81	0.85
Baseline Method-2	0.32	0.24	1.71	0.43
**Our Method**	0.24	0.15	1.32	0.25

## Data Availability

Data are contained within the article.
